# Changing the spatial pattern of *TFL1* expression reveals its key role in the shoot meristem in controlling *Arabidopsis* flowering architecture

**DOI:** 10.1093/jxb/erv247

**Published:** 2015-05-27

**Authors:** Kim Baumann, Julien Venail, Ana Berbel, Maria Jose Domenech, Tracy Money, Lucio Conti, Yoshie Hanzawa, Francisco Madueno, Desmond Bradley

**Affiliations:** ^1^John Innes Centre, Colney, Norwich NR4 7UH, UK; ^2^Instituto de Biología Molecular y Celular de Plantas (IBMCP), Consejo Superiorde Investigaciones Científicas (CSIC) – Universidad Politécnica de Valencia (UPV), Valencia 46022, Spain; ^3^Department of Crop Sciences and Institute for Genomic Biology, Affiliate in Department of Plant Biology, University of Illinois at Urbana-Champaign, 259 Edward R Madigan Lab, MC-051. 1201W Gregory Drive, Urbana, IL 61801, USA; ^4^Dipartimento di Bioscienze, Universita degli studi di Milano, Via Celoria 26, 20133, Milano, Italy

**Keywords:** Architecture, expression, flowering, identity, meristem, TFL1.

## Abstract

Models for the control of above-ground plant architectures show how meristems can be programmed to be either shoots or flowers. Molecular, genetic, transgenic, and mathematical studies have greatly refined these models, suggesting that the phase of the shoot reflects different genes contributing to its repression of flowering, its vegetativeness (‘veg’), before activators promote flower development. Key elements of how the repressor of flowering and shoot meristem gene *TFL1* acts have now been tested, by changing its spatiotemporal pattern. It is shown that *TFL1* can act outside of its normal expression domain in leaf primordia or floral meristems to repress flower identity. These data show how the timing and spatial pattern of *TFL1* expression affect overall plant architecture. This reveals that the underlying pattern of *TFL1* interactors is complex and that they may be spatially more widespread than *TFL1* itself, which is confined to shoots. However, the data show that while *TFL1* and floral genes can both act and compete in the same meristem, it appears that the main shoot meristem is more sensitive to *TFL1* rather than floral genes. This spatial analysis therefore reveals how a difference in response helps maintain the ‘veg’ state of the shoot meristem.

## Highlight

Plants carefully control where and when flowers are made through activators and repressors. We show that spatially the shoot meristem is key in responding to the repressors of flowering *TFL1*.

## Introduction

The development of plants with different architectures reflects variation in underlying molecular patterns (Sussex and Kerk, 2001). These patterns are complex interactions of gene, protein, and metabolite systems (Kaufmann *et al.*, 2010a; Sparks *et al.*, 2013; Park *et al.*, 2014). Analysis of these systems has identified many of the genes that control the formation of parts, their identity, position, and complexity (Benlloch *et al.*, 2007; Studer *et al.*, 2011; Alonso-Blanco and Mendez-Vigo, 2014). Such phenotypic traits have been selected during evolution and characterize different species, but, for any gene, what elements of its pattern are key in giving rise to a particular architecture?

Pattern elements include the level of gene expression, its timing, and in which cells it is expressed. For example, as plants pass through various developmental stages, different genes are expressed at appropriate levels and times, such as those maintaining the vegetative state of *Arabidopsis* (Poethig, 2010; Andres and Coupland, 2012). Other genes are expressed in specific domains to direct formation of organs in particular places, such as petals in flowers (O’Maoileidgh *et al*., 2014). The diversity of forms amongst species is the result of the evolution of these complex patterns. In addition to representing where, when, or how much of a gene is expressed, these patterns also determine potential new interacting genetic networks. Analysis of gene interactions, expression patterns, and loss- or gain-of-function phenotypes give us models for how these systems might operate. However, central to any models is the need to test how any pattern element contributes to generating a particular form. What is the effect of changing an element so a gene is expressed in novel domains or with different timing or levels? These questions have now been addressed for *TFL1*, a controller of plant architecture (Shannon and Meeks-Wagner, 1991; Alvarez *et al.*, 1992; Schultz and Haughn, 1993; Ohshima *et al.*, 1997).

*TFL1* functions as a repressor of flowering and belongs to a small family of six genes in *Arabidopsis* (Kim *et al.*, 2013). *FT* is a member of this family and is a key promoter of flowering, a florigen (Kardailsky *et al.*, 1999; Kobayashi *et al.*, 1999; Abe *et al.*, 2005; Wigge *et al.*, 2005; Shalit *et al.*, 2009). The antagonism, different levels, and different expression patterns of these very similar proteins affect overall plant architecture (Hanzawa *et al.*, 2005; Wigge *et al.*, 2005: Shalit *et al.*, 2009; Jaeger *et al.*, 2013; Ho and Weigel, 2014). *TFL1* can act through transcription to repress floral genes, and can modulate protein cellular protein trafficking patterns (Sohn *et al.*, 2007; Hanano and Goto, 2011). Controlling the pattern and levels of *TFL1* interactions in relation to floral meristem genes is predicted to be crucial (Prusinkiewicz *et al.*, 2007; Koes, 2008; Jaeger *et al.*, 2013; Park *et al.*, 2014).

In wild-type (WT) *Arabidopsis*, *TFL1* expression is limited to shoots (Simon *et al.*, 1996; Bradley *et al.*, 1997; Ratcliffe *et al.*, 1999). During the vegetative phase, *TFL1* is weakly expressed in the centre of the shoot meristem. This vegetative shoot meristem generates leaf primordia from its flanks to form a compact rosette. Upon integration of developmental and environmental signals, the shoot meristem makes cauline leaves (CLs; bearing shoot meristems in their axils) and the shoot elongates (bolts) (Poethig, 2010; Andres and Coupland, 2012; O’Maoileidgh *et al*., 2014). The level of *TFL1* expression is up-regulated at this stage. *TFL1* expression remains high in the shoot meristem as it generates floral meristems from its flanks, and *TFL1* becomes strong in the stem.

This pattern of *TFL1* expression appears to reflect its function. In *tfl1* mutants, the shoot meristem makes fewer rosette leaves (RLs; see Table 1 for abbreviations) and plants bolt early compared with the WT (Shannon and Meeks-Wagner, 1991; Schultz and Haughn, 1993). Also, *tfl1* mutants make fewer CLs and only a few flowers (Fs) before the shoot meristem converts to a floral meristem to give a terminal flower (Shannon and Meeks-Wagner, 1991; Alvarez *et al.*, 1992; Schultz and Haughn, 1993). Thus *TFL1* is needed to maintain and regulate shoot identity throughout the different phases of the plant life cycle to generate a particular architecture.

**Table 1.** Abbreviations used for growth phases and plant organs scored

Phase	Phase abbreviation	Lateral organs made by shoot meristem	Organ abbreviation
Vegetative rosette	V	Leaves	L
Inflorescence bolting and bearing cauline leaves	I1	Cauline leaves	CL
Inflorescence/*ap1*-like structures without subtending leaves	I1*	Shoots or *ap1*-like flowers without subtending cauline leaves	I1* shoots, *ap1*-like F
Inflorescence with flowers	I2	Flowers	F

Models suggest how the *TFL1* expression pattern controls *Arabidopsis* architecture (Ratcliffe *et al.*, 1999; Liljegren *et al.*, 1999; Ferrandiz *et al.*, 2000; Prusinkiewicz *et al.*, 2007). *TFL1* delays the action of floral signals at the shoot meristem that promotes bolting, and *TFL1* prevents their activity in the shoot meristem so that floral genes are not expressed in the shoot but only in lateral meristems. This maintains shoot identity and prevents the shoot meristem converting to a flower. An integrated model summarizes these interactions as acting upon the vegetativeness character (‘veg’) of the shoot apex (Prusinkiewicz *et al.*, 2007). *TFL1* contributes to ‘veg’ to delay flowering. Models also show how genes affecting floral meristem development, such as *LFY*, *AP1*, *CAL*, or *FUL*, prevent *TFL1* expression in floral meristems. For example, both *LFY* and *AP1* repress *TFL1* by direct binding to its promoter (Kaufmann *et al.*, 2010b; Winter *et al.*, 2011). Also, a series of MADS box transcription factors promoting floral meristem identity suppress *TFL1* in emerging floral meristems in *Arabidopsis*, and similarly in other species (Liu *et al.*, 2013). This mutual inhibition results in clear domains of expression and activity, and a shoot architecture of leaves and branches at the base and an elongated stem with flowers on its sides. These models are consistent with mutant phenotypes and the expression patterns of these genes in various backgrounds (Blazquez *et al.*, 2006). However, how do these myriad of interactions tie in with the spatial network of *TFL1* action?

These models are supported by the phenotypes of plants ectopically expressing floral genes or *TFL1* (Weigel and Nilsson, 1995; Mandel and Yanofsky, 1995; Ratcliffe et al., 1998, 1999; Liljegren *et al.*, 1999; Parcy *et al.*, 2002). Most of these ectopic studies have used the *p35S* promoter which is expressed constitutively, in most tissues, though patterns can vary (van Leeuwen *et al*., 2001). In *p35S*::*LFY* or *p35S*::*AP1* plants, all phases are shorter (like *tfl1* mutants), with plants bolting early and shoot meristems converting to flowers (Mandel and Yanofsky, 1995; Weigel and Nilsson, 1995). In *p35S*::*TFL1* plants, all phases are longer, with more RLs and CLs. These plants also make a novel I1* phase of shoots without subtending CLs and *ap1*-like structures (Ratcliffe *et al.*, 1998). In double transgenics such as *p35S::pAP1;35S::TFL1*, intermediate phenotypes occur, again suggesting that *TFL1* and floral genes are antagonistic in their effects on meristem identity (Liljegren *et al.*, 1999; Ratcliffe *et al.*, 1999). However, the *p35S* promoter does not tell us where these genes act; is it in the same or different tissues? Neither does *p35S* tell us how genes may have quantitative effects or whether their expression at different times has different effects on architecture.

In this study, tests were carried out to determine how different elements of the *TFL1* expression pattern contribute to plant architecture. Three different promoters were used to change the regulation of *TFL1* expression, and when and where it can act. Further, by using floral promoters to express *TFL1*, it was directly tested how floral genes and *TFL1* compete in the same tissues, and at the same time. By using *tfl1* and WT backgrounds, the action of *TFL1* in the shoot meristem could also be directly compared with its effects in lateral meristems, using the same constructs. It is shown that *TFL1* can act outside of the shoot meristem, affecting the fate of lateral primordia. Therefore, *TFL1* interactors and signalling components to affect ‘veg’ are not restricted to the shoot meristem. *TFL1* prevented leaves from becoming flowers and delayed floral gene action. However, floral genes eventually overcame *TFL1* action in lateral meristems. Despite ectopic expression, plants can tolerate quite different *TFL1* expression patterns and yet still generate a raceme. The use of different spatial promoters has allowed the suggestion that *TFL1* is expressed in its specific pattern to engineer sharp transitions from shoots to flowers, with the main and lateral meristems having different responses at the flowering transition.

## Materials and methods

### Plant materials and analyses

*Arabidopsis* ecotype Col (WT) and *tfl1-1* (Shannon and Meeks-Wagner, 1991) were used as controls and hosts for transformation of promoter::*TFL1* constructs. Lines were compared with *p35S*::*TFL1* and grown in the greenhouse under controlled temperatures of 20–28 °C and long-day (LD) photoperiods supplemented with light as necessary [400W Philips HDK/400 HPI (R) (N) or cool light from fluorescent tubes at an intensity of 90–120 mmol m^–2^ s^–1^] to give 16h light/8h dark as described (Ratcliffe *et al.*, 1998).

The WT and *tfl1* mutants were transformed with *pLFY::TFL1* or *pAP1::TFL1*, and 14–33 transformants were obtained. These transformants were analysed and 7–12 single insertion locus lines were identified for each construct. For *pANT::TFL1*, only WT plants were directly transformed. Subsequently, lines were used to introduce *pANT::TFL1* into *tfl1* mutants by crossing. Five to seven lines for all constructs were preliminarily analysed to show consistent results (Supplementary Fig. S1 available at *JXB* online). Data from two representative strong lines for each construct were collected when all lines were grown at the same time and under the same LD greenhouse conditions, allowing a direct, quantitative comparison of phenotypes. Lines were sown and analysed in 3–5 independent experiments, and the results obtained showed that the phenotypes of all lines were highly consistent relative to controls. Abbreviations used for phenotypes of growth phases and plant organs scored are given in Table 1.

### Promoter*::TFL1* constructs

The *ANT* promoter was a 4.2kb 5’ region (pYM-94-1) kindly provided by Yukiko Mizukami (Grandjean *et al.*, 2004). The *LFY* promoter was a 2.3kb 5’ region (pDW132) kindly provided by Detlef Weigel (Blazquez *et al.*, 1997). The *AP1* promoter was a 1.7kb 5’ region (pKY72) kindly provided by Marty Yanofsky (Hempel *et al.*, 1997). The *TFL1* cDNA (Hanzawa *et al*, 2005) was amplified with primers Y34 (AGTGGATCCATGGAGAATATGGGAACT) and Y37 (ATGGAATTCCTAGCGTTTGCGTGCAG) to add *Xho*I and *Bgl*II sites 5’ of ATG and 3’ of the stop codon, respectively, and cloned into pGEM-T (Stratagene). This *Xho*I–*Bgl*II fragment was cloned into a vector with a multiple cloning site and the *p35S* terminator to give pK6. The different promoter fragments were cloned into this vector as *Xho*I–*Bgl*II (*pANT*), *Sal*I–*Bam*HI (*pLFY*), or *Hin*dIII–*Bam*HI (*pAP1*), respectively. The full promoter*::TFL1* fragments were then transferred as *Xho*I–*Bam*HI (*pANT::TFL1*), *Xho*I–*Bam*HI (*pLFY::TFL1*), or *Hin*dIII–*Bam*HI (*pAP1::TFL1*) to the binary vector pGreen0229 (Basta resistant; Hellens *et al.*, 2000). This gave pK31 (*pANT::TFL1*), pK30 (*pLFY::TFL1*), and pK26 (*pAP1::TFL1*), which were transformed into *Agrobacterium* GV3101 with pSOUP and used to transform *Arabidopsis* plants by dipping as described (Clough and Bent, 1998).

### RNA *in situ* hybridization

RNA *in situ* hybridization experiments with *TFL1*, *LFY*, *AP1*, and *ANT* antisense and sense probes were carried out as described (Ferrandiz *et al.*, 2000). Note that quantification of *in situ* signals is not possible. Signal from probes cannot be compared to say if one is at different levels of expression, even though all probes are made at the same time, in the same way, as their base sequences. Also, the same probe on two different plants is still difficult to compare as tissue fixation and tissues vary.

## Results

Three promoters, *pANT*, *pLFY*, and *pAP1*, were used to express *TFL1* in novel patterns during *Arabidopsis* development. These allowed *TFL1*, normally expressed only in the centre of shoot meristems, to be expressed ectopically in leaf primordia and floral meristems. In the WT, *pANT* is expressed in leaf primordia on the flanks of the shoot during all phases of growth (Elliott *et al.*, 1996; Klucher *et al.*, 1996; Long and Barton, 2000; Mizukami and Fischer, 2000; Grandjean *et al.*, 2004). *pLFY* is weakly expressed in leaf primordia, but strongly in young floral meristems (Blazquez *et al.*, 1997; Hempel *et al.*, 1997). *pAP1* is only expressed in floral meristems, from stage 1 (Mandel *et al*., 1992; Hempel *et al.*, 1997). The promoter fragments used have been shown largely to direct these expression patterns (Hempel *et al.*, 1997; Krizek 1999; Benlloch *et al.*, 2011). Further, the data below also showed that these promoters could drive expression of *TFL1* ectopically in such patterns. In the *tfl1* mutant background, both *pLFY* and *pAP1* become ectopically active in the shoot meristem (Weigel *et al.*, 1992; Bowman *et al.*, 1993; Gustafson-Brown *et al.*, 1994; Bradley at al., 1997; Liljegren *et al.*, 1999). The *35S* promoter (*p35S*) was also used as a control to drive general, constitutive *TFL1* expression in the WT during all phases and in most tissues as described (Ratcliffe *et al*., 1988). This promoter is considered strong and general, but can be variable, especially due to position effects (van Leeuwen *et al*., 2001).

Abbreviations were used in scoring phenotypes in terms of growth phases and types of organs generated from lateral primordia and meristems (Table 1).

### Different promoters complement the *TFL1* vegetative phase defect

The effects of the promoter*::TFL1* constructs on vegetative development were investigated in terms of whether they extend the vegetative phase so that more RLs were generated.

**Fig. 1.**
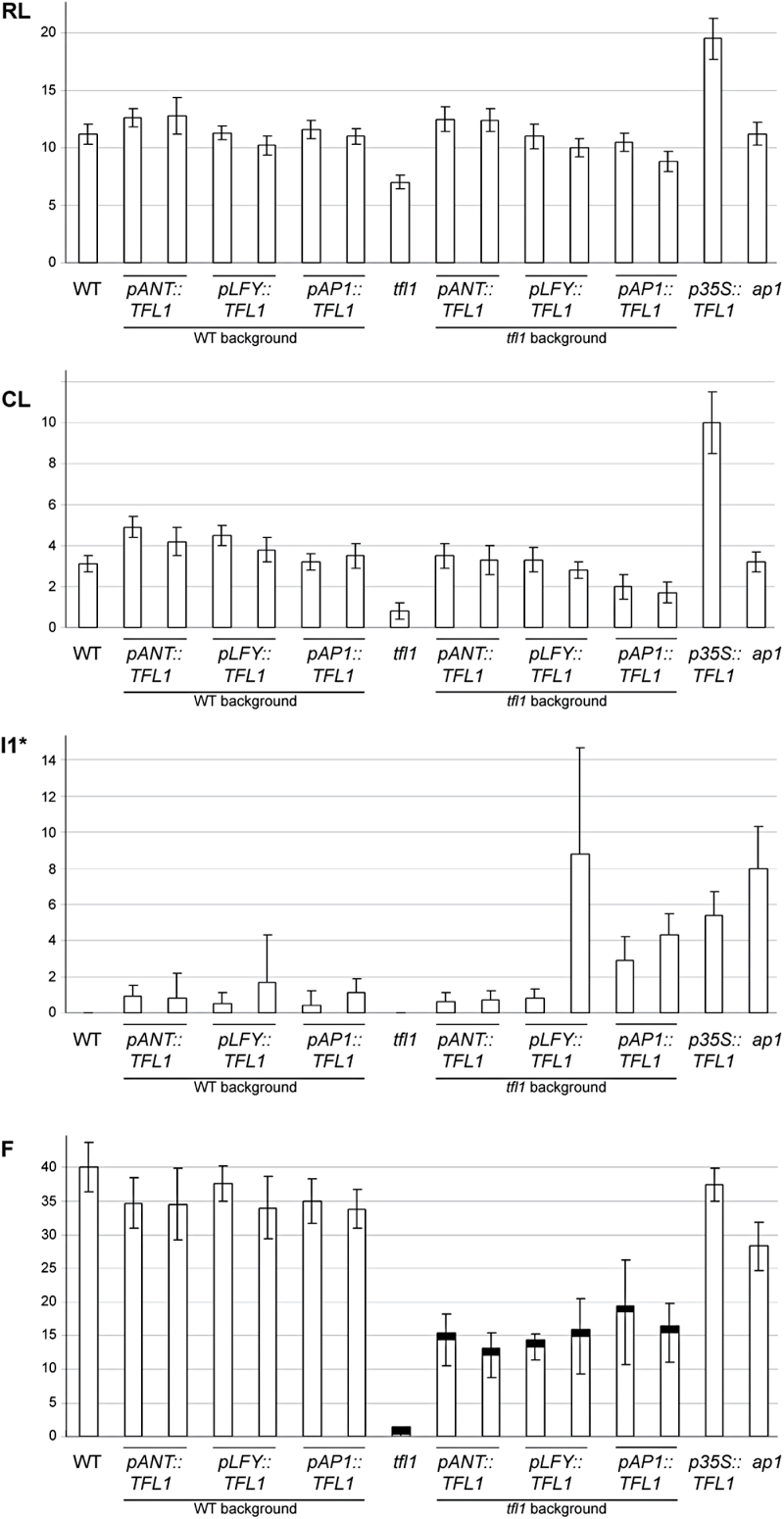
Ectopic *TFL1* affects plant organ numbers. The number of rosette leaves (RLs), cauline leaves (CLs), I1* structures (shoots without subtending CLs or *ap1*-like structures), and flowers (Fs) made by the main shoot were recorded for wild-type (WT) *Arabidopsis* or *tfl1-1* mutants containing *pANT::TFL1*, *pLFY::TFL1*, or *pAP1::TFL1*. WT plants containing *p35S:*:*TFL1* and *ap1-12* mutants were also analysed. Numbers represent the average of 23–54 plants with standard deviations as shown. The solid black bars in (F) in the *tfl1* background represent termination of the main shoot by conversion to a flower.

WT plants made ~11 RLs in long days (Fig. 1). No significant changes in leaf number were observed in any lines carrying any of the three constructs (Fig. 1). The range of leaf numbers was greater in plants carrying *pANT::TFL1* (9–16 leaves compared with 10–13 for the WT), but the averages were not statistically significant. This variability for *pANT::TFL1* was seen in different experiments. In contrast, plants carrying *p35S::TFL1* (which is strongly expressed in all tissues, including primordia and the shoot meristem) had an extension of the vegetative rosette (V) phase to 19 RLs, as previously shown (Fig. 1; Ratcliffe *et al.*, 1998).

*TFL1* extended the V phase in the WT, but only when expressed via *p35S*. There are a number of differences between *p35S* and the other promoters, including the timing and expression domain. To determine which aspect of *p35S* was important, either other promoters that had each of these features could be sought or these same promoters could be used but the genetic background could be altered to change their pattern. It was possible to change the pattern of these tested promoters by putting them into the *tfl1* mutant background. In *tfl1*, *pLFY* and *pAP1* express *TFL1* in primordia and throughout the shoot meristem (a change in domain), and earlier than in the WT (change in timing).

**Fig. 2.**
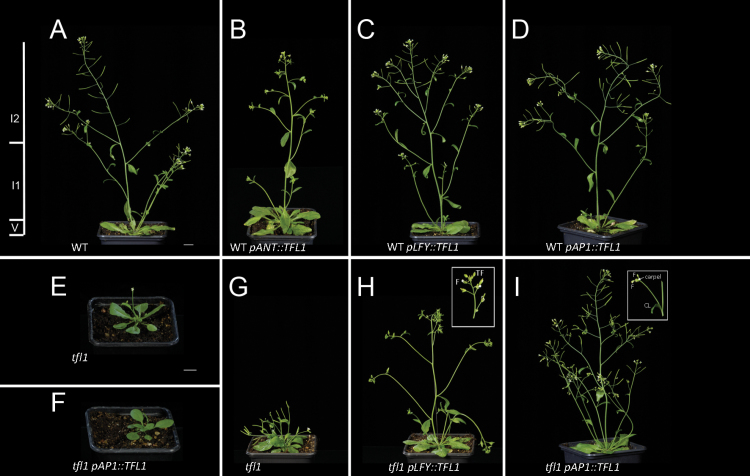
Plant architectures due to *TFL1* expression. (A–D) Mature plants of *Arabidopsis* WT (A) or WT containing *pANT::TFL1* (B), *pLFY::TFL1* (C), or *pAP1::TFL1* (D). In (A), the WT phases V, I1, and I2 are indicated. (E, F) Young *tfl1-1* mutant plants already bolted with terminal flowers (E) compared with *tfl1* containing *pAP1::TFL1* at the same age of 16 d (F). (G–I) Mature plants showing *tfl1-1* (G) or *tfl1-1* containing *pLFY::TFL1* (H) or *pAP1::TFL1* (I). the insert in (H) shows that these plants eventually make normal flowers and terminate. Insert in (I) shows CLs with axillary *ap1*-like structures. Scale bars=1cm.

Analysis of *pANT::TFL1* or *pLFY::TFL1* in the *tfl1* mutant showed that the V phase was extended compared with *tfl1* (Fig. 1). The *tfl1* mutant had a shorter V phase compared with transformants (Fig. 2E, F). After ~16–20 d, the *tfl1* mutant had already flowered and made seed pods (siliques). At this time point, *tfl1* lines carrying *pLFY::TFL1* or *pAP1::TFL1* had just started to bolt and were making flowers that had not yet matured. The common effect in all lines was to restore the V phase to WT (Fig. 1). Interestingly, *pAP1::TFL1* could also restore the V phase to WT in the strongest examples, and weaker lines always made significantly more RLs than *tfl1*. Unlike *p35S*, all other promoter lines made WT numbers of RLs, not more.

**Fig. 3.**
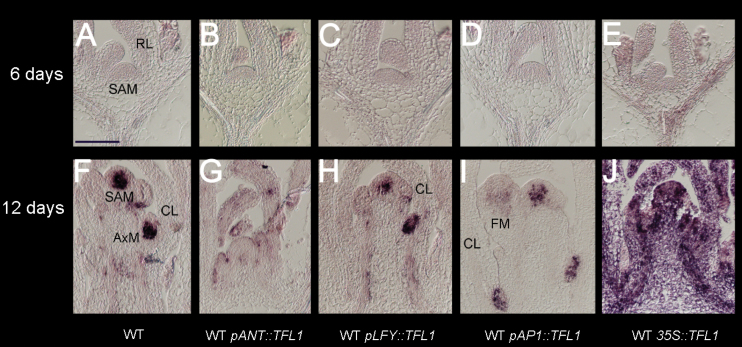
Early *TFL1* expression patterns in the WT background. (A–E) *TFL1* expression in the vegetative phase (6-day-old plants) of the WT (A) or the WT containing *pANT::TFL1* (B), *pLFY::TFL1* (C), *pAP1::TFL1* (D), or *p*35S::*TFL1* (E). For example, in (A), the shoot apical meristem (SAM) and rosette leaves (RL) generated by this meristem are indicated. (F–J) *TFL1* expression in early to late I1 phase (10- to 12-day-old plants) of the WT (F) or the WT containing *pANT::TFL1* (G), *pLFY::TFL1* (H), *pAP1::TFL1* (I), or *p35S*::*TFL1* (J). Examples of inflorescence SAM, axillary meristems (AxM) in axils of cauline leaves (CL) and floral meristems (FM) are highlighted. All images were obtained with the same probes and signals developed for the same time. Signal is seen as a purple stain on a pale/pink background. Scale bar=100 μm.

The expression patterns of *TFL1* were analysed in the different lines by RNA *in situ* hybridization to see how they related to the plant phenotypes. In the vegetative phase of all WT lines, no early endogenous or transgenic *TFL1* expression was clearly seen, except for general, constitutive *p35S::TFL1* (Fig. 3A–E). Thus the lack of any *TFL1* effect on the vegetative phase was most probably simply a lack of detectable expression. This may reflect the use of promoter fragments, sensitivity of detection, or even mRNA stability at early stages.

**Fig. 4.**
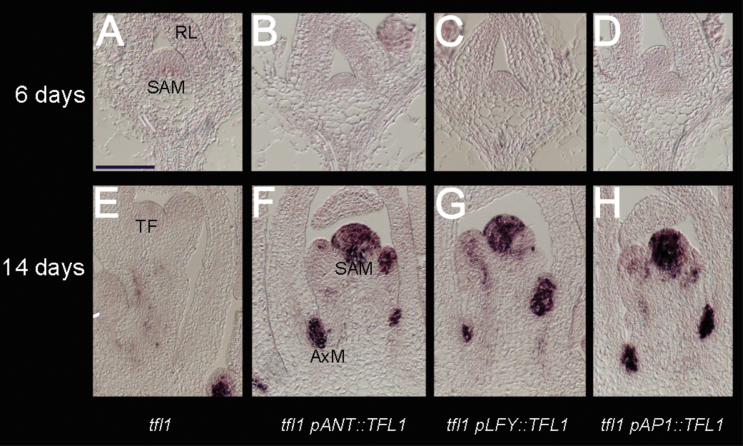
Early *TFL1*/*tfl1-1* expression patterns in the *tfl1-1* background. (A–D) *TFL1*/*tfl1-1* expression at 6 d in *tfl1-1* (A) or *tfl1-1* containing *pANT::TFL1* (B), *pLFY::TFL1* (C), or *pAP1::TFL1* (D). The shoot apical meristem is generating rosette leaves (RL). (E–H) *TFL1*/*tfl1-1* expression at 12–14 d in the I1–I2 phase of *tfl1-1* (E) or *tfl11-1* containing *pANT::TFL1* (F), *pLFY::TFL1* (G), or *pAP1::TFL1* (H). In *tfl1*, the SAM has already converted to a terminal flower (TF) while the other lines have *TFL1/tfl1-1* mRNA in the shoot apical meristems but are still not terminating at this stage. All images were obtained with the same probes and signals developed for the same time. Signal is seen as a purple stain on a pale/pink background. Scale bar=100 μm.

In the *tfl1* mutant background, no endogenous mutant *tfl1-1* mRNA was seen at the early phase (Fig. 4A). Similarly, no transgenic *TFL1 or tfl1-1* signal (together referred to as *TFL1/tfl1-1*) was seen at early time points, suggesting that it was below the detection limit (Fig. 4A–D). Thus, despite undetectable expression, all promoter*::TFL1* lines in *tfl1* had V phases restored to WT. Therefore, *tfl1* may be easily complemented by different promoters, but the length of the V phase may be generally robust due to many flowering pathways controlling the ‘veg’ character of this growth phase (Prusinkiewicz *et al.*, 2007).

### Increased cauline leaf numbers by ectopic *TFL1*

After the V phase, the *Arabidopsis* shoot enters the first inflorescence (I1) phase, making CLs that have secondary shoots in their axils, on an elongated stem (bolt). WT plants made about three CLs on the main stem (Figs 1, 2). In the WT background, ectopic expression of *TFL1* during this phase increased the number of CLs (Fig. 1). The strongest lines carrying *pANT::TFL1* and *pLFY::TFL1* showed an increase of 1–2 CLs compared with the WT. The range of CL numbers was only 2–4 for the WT, but 2–7 for *pANT::TFL1*. Therefore, these transformed plants displayed a more branching architecture compared with the WT (Fig. 2A–C). In contrast, WT lines containing *pAP1::TFL1* had no change in their numbers of CLs and appeared as WT in I1 (Figs 1, 2D). Plants carrying *p35S::TFL1* had a dramatic increase in the length of this phase (Fig. 1).

The *tfl1* mutants made only one CL compared with three in the WT (Figs 1, 2). Also, in *tfl1* mutants, all CLs had single flowers in their axils, while WT plants had shoots (Fig. 2A, E, G). In the *tfl1* background, *pANT::TFL1* and most *pLFY::TFL1* lines had CL numbers similar to the WT (Fig. 1). Also, these lines had shoots in their CL axils (Fig. 2H). In *tfl1*, *pAP1::TFL1* did not restore CL numbers to WT, but their numbers were significantly increased in the strongest lines compared with *tfl1* (Fig. 1). Also, for the stronger lines, CLs usually had shoots in their axils (Fig. 2I). There was only one exception (of a flower) in 146 individuals scored. For the weaker lines, CLs often had axillary shoots, but flowers or *AP1*-like flowers (see below) were found at frequencies of 25–45% (Fig. 2I insert).

For plants in the I1 phase, endogenous WT *TFL1* expression was seen for all lines in the shoot meristems (Fig. 3F–I). In the WT, *TFL1* mRNA was observed in the main shoot meristem and stem tissues, and in the axillary shoot meristems of CLs (Fig. 3F). A similar pattern was seen for the different lines, but each line had a different pattern of ectopic *TFL1* expression superimposed on the endogenous *TFL1* mRNA pattern. For *pANT::TFL1*, ectopic *TFL1* expression was seen in CLs (Fig. 3G). In *pLFY::TFL1* there was also *TFL1* expression in CLs, while *pAP1::TFL1* lines had no *TFL1* mRNA in leaves, only hints of ectopic expression in the first floral meristems as plants entered I2 (Fig. 3H, I). The *p35S::TFL1* lines showed expression throughout most tissues (Fig. 3J). Therefore, these expression patterns appeared to correlate with small effects on the I1 phase for *pANT* and *pLFY*, as ectopic *TFL1* mRNA appeared in CLs. No I1 expression was observed for *pAP1*, in agreement with no phenotypic effect in this phase. In contrast, the general expression of *p35S* must account for its strong I1 phenotype. It is difficult to comment on levels of expression as plant tissues differ; however, *in situ* hybridizations do reveal the distribution, and this is clearly more extensive in *p35S*. Thus making more CLs and branching architecture is dependent upon the pattern of *TFL1* expression. This must then contribute to maintaining the ‘veg’ character to delay phase transitions (Prusinkiewicz *et al.*, 2007). It suggests that repressors such as TFL1 can contribute to ‘veg’ even when expressed ectopically outside of the shoot meristem.

During this I1 phase in *tfl1* mutants, endogenous mutant *tfl1-1* mRNA was absent from the main shoot as it had already converted to a terminal flower by 12–14 d (Fig. 4E). Signal was restricted to young axillary meristems in the axils of RLs that had not yet converted to terminal flowers (Fig. 4E). For the different promoter lines, *TFL1/tfl1-1* mRNA was seen for much longer in the main shoot meristems (Fig. 4F–H). Also, signal was strong in lateral meristems of CLs, correlating with their conversion from axillary flowers to shoots in these lines. It was also seen that *TFL1/tfl1-1* signal appeared to be more extensive throughout the shoot meristem, not as restricted to the centre as in the WT. This novel pattern may reflect the partial floral nature of the main meristem to allow these promoters to be expressed beyond the normal central domain of *TFL1*. How each domain within the meristem contributes to the effect of *TFL1* on ‘veg’ and delaying flowering cannot be resolved here.

### *TFL1* inhibits floral meristem development

After the I1 phase of making CLs, WT plants enter a second inflorescence phase (I2) and the shoot meristem generates floral meristems (FMs) from its flanks. These FMs proceed through various stages of development until forming siliques (Fig. 2A; Smyth *et al.*, 1990). Expression of *LFY* from stage 0, and *AP1* from stage 1, reflecting low ‘veg’, ensures suppression of shoot identity and production of flowers.

In all the transgenic lines tested here, expression of *TFL1* from *pANT*, *pLFY*, or *pAP1* inhibited floral meristem development, delaying the production of flowers and causing the production of an I1* phase. This phase was also seen in *p35S::TFL1* lines, and consisted of shoot-like structures without any subtending CL, or *ap1*-like flowers that resulted in multiple siliques arising from a common floral stem, the pedicel (e.g. Fig. 2 insert). Although numbers were variable, these structures were found in all transgenic lines (Fig. 1). In contrast, these structures were rarely seen in control WT or *tfl1* plants (Fig. 1; zero in this experiment). Although clear in the WT background, the number of novel structures generated was small (0.1–2) for any of the promoters used (Fig. 1). Stronger effects appeared in the *tfl1* mutant background, where both *pLFY::TFL1* and *pAP1::TFL1* gave up to 4–8 structures.

The length of I1* seen in *pAP1::TFL1* and *pLFY::TFL1* lines was sometimes the same as in *p35S::TFL1* (Fig. 1). Also, *pLFY::TFL1* usually gave I1* shoots before I1* *ap1*-like floral structures, while *pAP1::TFL1* rarely gave I1* shoots and more usually gave only *ap1*-like floral structures. *ap1* mutants were examined, and it was found that, as in previous reports, an early, strong phenotype could be distinguished where mutant plants made structures similar to the *ap1*-like floral structures recorded in the transgenic lines, with flowers within flowers (Figs 1, 2I insert; Bowman *et al.*, 1993). Later, weak phenotypes were observed where flowers were abnormal (e.g. reduced petals), but did not have flowers within flowers. These later weak phenotypes were called *ap1*-like ‘flowers’ for this comparative analysis, as the final siliques were singular, as in the WT, rather than having multiple siliques on one pedicel. Thus the strongest *pAP1::TFL1* lines could have an I1* phase similar to *ap1* mutants (Fig. 1).

The I1* phase of *pANT::TFL1* lines consisted of both I1* shoots and *ap1*-like flowers, but this phase in *tfl1* was short as in the WT (Fig. 1). Therefore, unlike *pLFY* or *pAP1*, the *pANT::TFL1* lines never appeared to have a strong effect on flower development.

Since all lines had significant delays in making the transition from CL production (I1) to normal flowers (I2) and made a I1* phase, *TFL1* expression was analysed in comparison with the floral genes *LFY* and *AP1*.

**Fig. 5.**
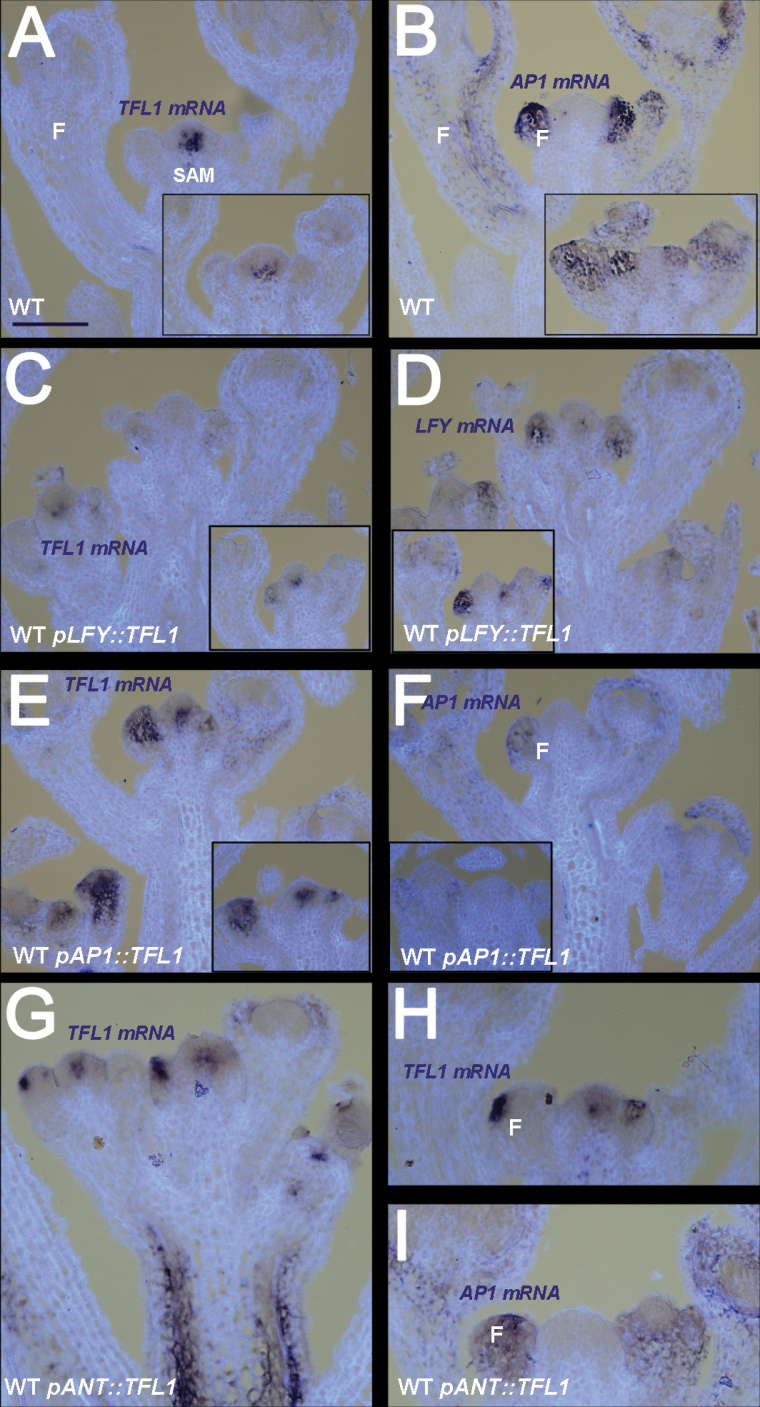
*TFL1* and floral gene expression patterns in inflorescences in the WT background. (A, B) WT flowering shoots in the I2 phase at 21 d showing expression of *TFL1* (A) or *AP1* (B). Inserts in TFL1 (A) and *LFY* (B) at 17 d. (C, D) WT plants containing *pLFY::TFL1* at 16 d showing *TFL1* (C) and *LFY* (D) expression. Inserts show another transgenic line at 21 d. (E, F) WT plants containing *pAP1::TFL1* at 21 d showing *TFL1* (E) and (*AP1*) expression. Inserts show another line. (G–I) WT plants containing *pANT::TFL1*. Expression of *TFL1* in a young tertiary shoot (G). Expression of *TFL1* in an older secondary shoot (H) and *AP1* expression (I). Shoot apical meristem (SAM), flower (F), and corresponding mRNA signals seen as a purple stain on pale blue/white tissue background. Scale bar=100 μm.

In the WT, *TFL1* was expressed in the centre of the shoot meristem (and weakly in inflorescence stems), but not in lateral primordia or floral meristems (Fig. 5A). In a complementary manner, *LFY* and *AP1* were not expressed in the shoot meristem of the WT, but only in the lateral meristems of I2, the floral meristems (Fig. 5B).

In WT lines with *pLFY::TFL1*, *TFL1* was expressed ectopically and overlapping with *LFY* (Fig. 5C, D). Note that although *LFY* signal appeared stronger than *TFL1*, it could not be concluded that *TFL1* was expressed less or was less stable than *LFY*, as the probes were different. WT lines containing *pAP1::TFL1* also had clear ectopic expression of *TFL1* in lateral meristems (Fig. 5E). Endogenous *AP1* expression overlapped with *TFL1* (Fig. 5F). These patterns for *pLFY* and *pAP1* lines were consistent for many different lines, while lines with the weakest phenotypes had undetectable ectopic *TFL1* expression. Also, the patterns were generally consistent over 16–22 d of growth, during which time some I1* shoot or *ap1*-like structures would have been made, as well as normal flowers.

WT lines containing *pANT::TFL1* showed ectopic *TFL1* in the young developing lateral meristems and their primordia (Fig. 5G). Comparison of *TFL1* with *AP1* in these lines showed that ectopic *TFL1* occurred in lateral meristems that probably gave rise to flowers (Fig. 5H, I).

**Fig. 6.**
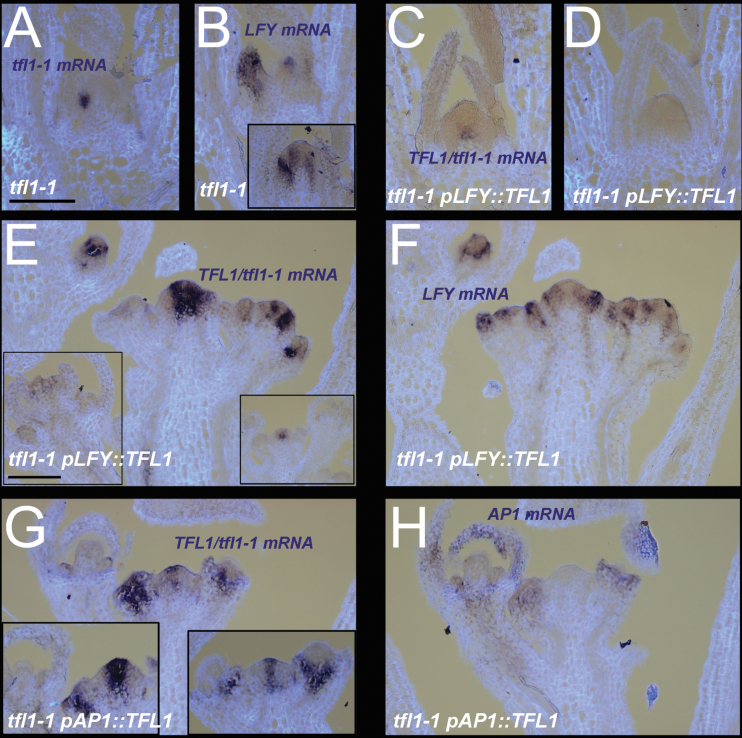
Expression patterns in *tfl1* mutant backgrounds. (A, B) Young 10-day-old *tfl1* mutants showing *tfl1-1* (A) and *LFY* (B) expression. The insert in (B) shows plants just starting to bolt and ectopically expressing *LFY* in the shoot. (C, D) Ten-day-old day *tfl1* plants containing *pLFY::TFL1* showing *TFL1*/*tfl1-1* (C) and absence of *LFY* (D) expression. (E, F) Older *tfl1* plants containing *pLFY::TFL1* at 17 d showing *TFL1*/*tfl1-1* (E) and *LFY* (F) expression. The left insert in (E) shows expression at 20 d in another line in an *ap1*-like structure. The right insert shows that *TFL1* expression is largely limited to the shoot meristem in *ap1* mutants. (G, H) At 21 d, *tfl1* mutants containing *pAP1::TFL1*, showing *TFL1*/*tfl1-1* (G) and *AP1* (H) expression. Inserts in (G) show other examples. Corresponding mRNA signals seen as a purple stain on pale blue/white tissue background. Scale bars=100 μm.

The *TFL1/tfl1-1* expression patterns were compared with those of *LFY* and *AP1*. Due to having common promoters, the pattern of *LFY* mRNA reflected the pattern of *TFL1* in *pLFY::TFL1* expression. In contrast, *tfl1-1* mRNA reflected its own promoter and this was highest in shoot-like structures. As *tfl1* mutant plants were just bolting, *tfl1-1* expression was seen in the main shoot (in the centre below the dome) at the same time as *LFY* also became ectopically expressed there (Fig. 6A, B). In *pLFY::TFL1*, the *TFL1/tfl1-1* RNA was also seen at bolting, but no clear expression was seen of *LFY* at this time, reflecting the delay in flowering (Fig. 6C, D). After bolting, *pLFY::TFL1* lines had *TFL1/tfl11-1* expression that partially overlapped with that of *LFY* (Fig. 6E, F). Ectopic *LFY* in the shoot meristem was weak compared with *LFY* in lateral meristems (Fig. 6F). Ectopic expression of *TFL1/tfl1-1* in the shoot meristem, beyond the central cells, was again seen. Some structures appeared *ap1*-like in tissue sections, and had ectopic *TFL1/tfl11-1* (Fig. 6E left insert). In contrast, in *ap1* mutant plants, *ap1* mutant flowers did not appear to have strong ectopic *TFL1* expression (Fig. 6E right insert).

For *pAP1::TFLl* lines in *tfl1*, ectopic expression of *TFL1* often appeared as clear as endogenous *tfl1-1* seen in the *tfl1* mutant itself (Fig. 6A, G). This expression was found at different time points, even when the shoot was making apparently normal flowers (Fig. 6G, H). Expression of *AP1* partially overlapped with ectopic *TFL1* in flowers in these lines (Fig. 6H). Interestingly, *AP1* was often undetectable in the shoot meristem, compared with lateral *AP1* (Fig. 6H). This suggested that *pAP1::TFL1* was also undetectable in the shoot meristem, yet no terminal flower was evident at any of these time points (13–21 d).

### Flowers are produced despite *TFL1* expression

For all of the lines in the WT background, normal-looking, fertile flowers (Fs) were produced after the I1* phase, typical of a wild-type I2 phase (Fig. 2A–D). Occasionally, *ap1*-like structures were found later on the shoot in I2. The inflorescences of WT and transgenic lines generated a similar number of flowers before normal senescence (Fig. 1). However, in one *pLFY::TFL1* line and one *pANT::TFL1* line, both of which had very weak V-I1* phenotypes, terminal flowers were made in I2 after ~25–30 flowers. This suggested problems in late *TFL1* function in these lines.

The I2 flowering phase of *tfl1* mutants is very short, with very few lateral Fs being made before the shoot meristem itself is converted to a terminal flower (Fig. 1). This gave *tfl1* mutant plants their characteristic short stature (Fig. 2E, G). In the *tfl1* mutant background, *pLFY::TFL1* and *pAP1::TFL1* lines made many more lateral Fs (up to 30 times) compared with *tfl1* (Fig. 1). These Fs also generally appeared normal, as in the WT, indicating that *LFY* and *AP1* were largely unaffected by co-expression of *TFL1* (Fig. 2H, I). However, during this same growth phase the conversion of the shoot meristem to terminal flowers was strongly delayed, indicating that *TFL1* strongly inhibited *LFY* and *AP1* action in the main shoot meristem, promoting the ‘veg’ character of these plants.

The conversion of the shoot meristem to a terminal flower results in a typical architecture of siliques clustered at the apex, with the central silique either normal or a bit smaller and distorted (Shannon and Meeks-Wagner, 1991; Schultz and Haughn, 1993). However, in ~5% of *pANT* and 20–25% of *pLFY* or *pAP1* lines, the apex appeared fasciated and bent as it terminated. This phenotype can often be seen in *lfy* mutants when they terminate in a carpel-like structure (Weigel *et al.*, 1992). This may reflect *TFL1* inhibiting LFY even at very late stages, still promoting ‘veg’.

## Discussion

Use of three independent promoters revealed how the timing and level of *TFL1* expression is important in affecting plant architecture. It was shown that *TFL1* is able to act outside of the shoot meristem. Ectopic expression of *TFL1* is sufficient to convert lateral meristems to leaves and shoots, by delaying the activity of co-expressed floral meristem identity genes. By expressing *TFL1* in different domains (using WT and *tfl1* backgrounds), it was also revealed that the main shoot appears more responsive to *TFL1* than lateral meristems, as more extensive phase effects were seen in the *tfl1* background where the promoters used became active not just in the lateral meristems but also in the shoot meristem. Therefore, the underlying spatiotemporal patterns of interactors for *TFL1* probably differ between shoot and lateral meristems. Thus *TFL1* promotes ‘veg’ most probably through the shoot meristem. These data should help in understanding which elements of the *TFL1* expression pattern are important in establishing and maintaining particular plant architectures.

### *TFL1* can function outside of the shoot meristem

Ectopic *TFL1* prevents lateral meristems from undergoing a floral fate. This change in pattern leads to increased branching and plant size, resulting in an altered architecture. In the WT, ectopic expression of *TFL1* (via *pANT* or *pLFY*) in lateral meristems resulted in more cauline leaves being made. The normal actions of *LFY*, and probably other factors such as *LIM1*, were inhibited by *TFL1* and so these factors were unable to act in their normal role to suppress leaf and shoot formation (Weigel *et al.*, 1992; Huala and Sussex, 1992; Saddic *et al.*, 2006). Leaf and shoot development occurred despite *LFY* being expressed at the same time and in the same place as *TFL1*. Further, when *LFY* action appeared to be partially restored (as cauline leaves were suppressed), *TFL1* still inhibited flower development in lateral meristems. The strongest effects gave rise to abnormal shoots similar to *ap1*;*lfy* double mutants, but more often to *ap1*-like structures (Bowman *et al.*, 1993; Weigel and Meyerowitz, 1993; Mandel and Yanofsky, 1995; Parcy *et al.*, 1998; Wagner *et al.*, 1999). Thus the action of both *LFY* and *AP1*, and probably other genes such as *FUL* or *CAL*, was inhibited by *TFL1* when co-expressed in the same primordia or meristems (Mandel and Yanofsky, 1995; Liljegren *et al.*, 1999; Ratcliffe *et al.*, 1999; Ferrandiz *et al.*, 2000).

*TFL1* may also act ectopically in the vegetative phase of *Arabidopsis* development. Of the promoters used, only *p35S* had a significant effect on RL number. As the other promoters are known to have only weak expression in early phases, which aspect of *p35S*, its high expression or expression in the shoot meristem and in leaves, was critical in affecting V phase, cannot be resolved.

A clear effect on the vegetative phase was found when *TFL1* was ectopic in both the lateral primordia and shoot meristem. In the *tfl1* mutant, both *pLFY::TFL1* and *pAP1::TFL1* increased the number of rosette leaves and delayed bolting. Both complemented the *tfl1* flowering time effect, and restored the number of RLs to WT, but not greater. There are two possibilities to explain why *TFL1* was now active in the vegetative phase. Either earlier expression of *TFL1* in the shoot is more effective in the vegetative phase, or a few lateral primordia are more sensitive to *TFL1* action, and so *TFL1* can act in lateral primordia which may be a characteristic of different subphases (Kersetter and Poethig, 1998; Hempel *et al*., 1998; Suh *et al.*, 2003). The first possibility is suggested to be more likely. First, the complementation of RL number in these lines was the same as when *TFL1* was only expressed in the shoot (as in WT plants). Secondly, *TFL1* has stronger effects (to inhibit floral genes) when expressed in the shoot meristem compared with lateral meristems (see below).

### Shoot and lateral meristems have different responses to *TFL1*

*TFL1* is more effective in inhibiting floral meristem genes when expressed in the main shoot. By introducing *pLFY::TFL1* or *pAP1::TFL1* into *tfl1*, *TFL1* became expressed in both lateral meristems and the shoot meristem, in direct competition with *LFY* and *AP1*. In the *tfl1* mutant, only one or two lateral flowers are made before the shoot itself is converted to a terminal flower. In *tfl1* carrying *pLFY::TFL1* or *pAP1::TFL1*, up to 30 lateral flowers were generated before *LFY* and *AP1* could finally overcome inhibition by *TFL1* and convert the shoot meristem into a terminal flower. Therefore, the competence of the shoot and lateral meristems differs for *TFL1* and *LFY*/*AP1* action, as, in these lines, all three genes are expressed together at the same level and with the same timing in the two types of meristem, lateral or shoot. This competence may reflect an underlying pattern of interactors needed for *TFL1*, or *LFY* and *AP1* action, to specify shoot or floral meristem identity. Potential interactors include bZIP transcription factors, one of which, *FD*, is expressed both in the shoot meristem and on its flanks in leaf and floral anlage (Abe *et al.*, 2005; Wigge *et al.*, 2005; Jaeger *et al.*, 2006).

The effects of ectopic *TFL1* on lateral meristem development were enhanced in the *tfl1* background. More I1*/*ap1*-like structures were made when *pLFY::TFL1* or *pAP1::TFL1* were active in *tfl1* compared with the WT. This may reflect earlier *TFL1* expression from these promoters in the shoot meristem so that *TFL1* affects the fate of the earliest cells (anlagen) destined to form the primordia. By establishing some *TFL1* in these cells, this might lead to greater inhibition of *LFY* and *AP1* when these cells emerge on the flanks of the shoot meristem. Therefore, even if *TFL1* is not expressed any more strongly than *LFY* or *AP1* in these lateral meristems, earlier expression (overlapping with key interactors) may be an important factor. The movement of TFL1 protein throughout the shoot meristem (which includes the anlagen) could restrict early floral gene effects (Conti and Bradley, 2007).

The present study also raises the important question of where or when *TFL1* cannot act. If *TFL1* was expressed later than the floral genes, what would happen? For example, *TFL1* expressed only in the later stages of FM development (via *pAG*) did not promote shoot development (Parcy *et al.*, 2002). Thus expression at the same time as *LFY* or *AP1* may be required to affect meristem identity. This is supported by studies on *ATC*, a *TFL1* homologue. *ATC* is expressed in the hypocotyl, and an *atc* mutant has no effects on meristem identity (Mimida *et al.*, 2001). However, if expressed via *p35S*, *ATC* can act as *TFL1*.

One idea of this work was to test if the indeterminate shoot architecture of *Arabidopsis* (a raceme) would be altered significantly. If the main shoot terminated in *tfl1*, but *TFL1* was expressed in the lateral meristem by *pANT*, for example, then maybe the lateral meristem would have grown and generated a new lateral meristem before terminating. If this occurred, then a branching determinate architecture could be formed, equivalent to the other major form of architecture, a determinate cyme. However, this did not happen by simply placing *TFL1* under the control of lateral/floral promoters in a *tfl1* mutant background. Rather, the data support models that predict it to be necessary to change both the pattern of *TFL1* and floral genes reciprocally (Prusinkiewicz *et al.*, 2007; Koes, 2008: Jaeger *et al.*, 2013). By changing promoters and thus gene regulation, interactions necessary to generate cyme architectures may result (Souer *et al.*, 2008; Kurokura *et al.*, 2013; Park *et al.*, 2014).

Studies in tomato on *TFL1* and *FT* homologues have used *p35S* and mutant backgrounds to highlight the differences in primary and axillary meristems in this species with a cymose architecture (Shalit *et al.*, 2009). In this case, it is suggested that balancing the levels of these factors has key effects on the fate of different meristem types.

## Supplementary data

Supplementary data are available at *JXB* online.

Figure S1. Independent lines show that ectopic *TFL1* affects plant organ numbers.

Supplementary Data
