# Supplementary material for: Changing the spatial pattern of TFL1 expression reveals its key role in the shoot meristem in controlling Arabidopsis flowering architecture
Source: J Exp Bot. 2015 May 27;66(15):4769–80. doi: 10.1093/jxb/erv247 (PMC4507777; doi:10.1093/jxb/erv247)
Supplement: Supplementary Data [file supp_66_15_4769__index.html]

Changing the spatial pattern of TFL1 expression reveals its key role in the shoot meristem in controlling Arabidopsis flowering architecture — Changing the spatial pattern of TFL1 expression reveals its key role in the shoot meristem in controlling Arabidopsis flowering architecture — Supplementary Data 

# Changing the spatial pattern of *TFL1* expression reveals its key role in the shoot meristem in controlling *Arabidopsis* flowering architecture

## Supplementary Data

Data files

- Supplementary Data - Supplementary Data
