# Supplementary material for: Changing the spatial pattern of TFL1 expression reveals its key role in the shoot meristem in controlling Arabidopsis flowering architecture
Source: J Exp Bot. 2015 May 27;66(15):4769–80. doi: 10.1093/jxb/erv247 (PMC4507777; doi:10.1093/jxb/erv247)
Supplement: Supplementary Data [file supp_erv247_jexbot143164_file001.pdf]

Journal of Experimental Botany

‘Changing the spatial pattern of *TFL1* expression reveals its key role in the shoot meristem to control Arabidopsis flowering architecture’

Kim Baumann[1], Julien Venail[1], Ana Berbel[2], Maria Jose Domenech[2], Tracy Money[1], Lucio Conti1[2], Yoshie Hanzawa[1,3], Francisco Madueno[2], Desmond Bradley[1]

Supplementary Figure S1. Independent lines show ectopic *TFL1* affects plant organ numbers

The number of rosette leaves (RL), cauline leaves (CL), I1* structures (shoots without subtending CL or *ap1*-like structures) and flowers (F) made by the main shoot were recorded for wild-type (WT) Arabidopsis or *tfl1-1* mutants containing *pANT::TFL1, pLFY::TFL1* or *pAP1::TFL1*. WT plants containing 35S::*TFL1* and *ap1*-12 mutants were also analysed. Numbers represent the average of 20-55 plants with standard deviations as shown. The solid black bars in (F) in the *tfl1* background represent termination of the main shoot by conversion to a flower.

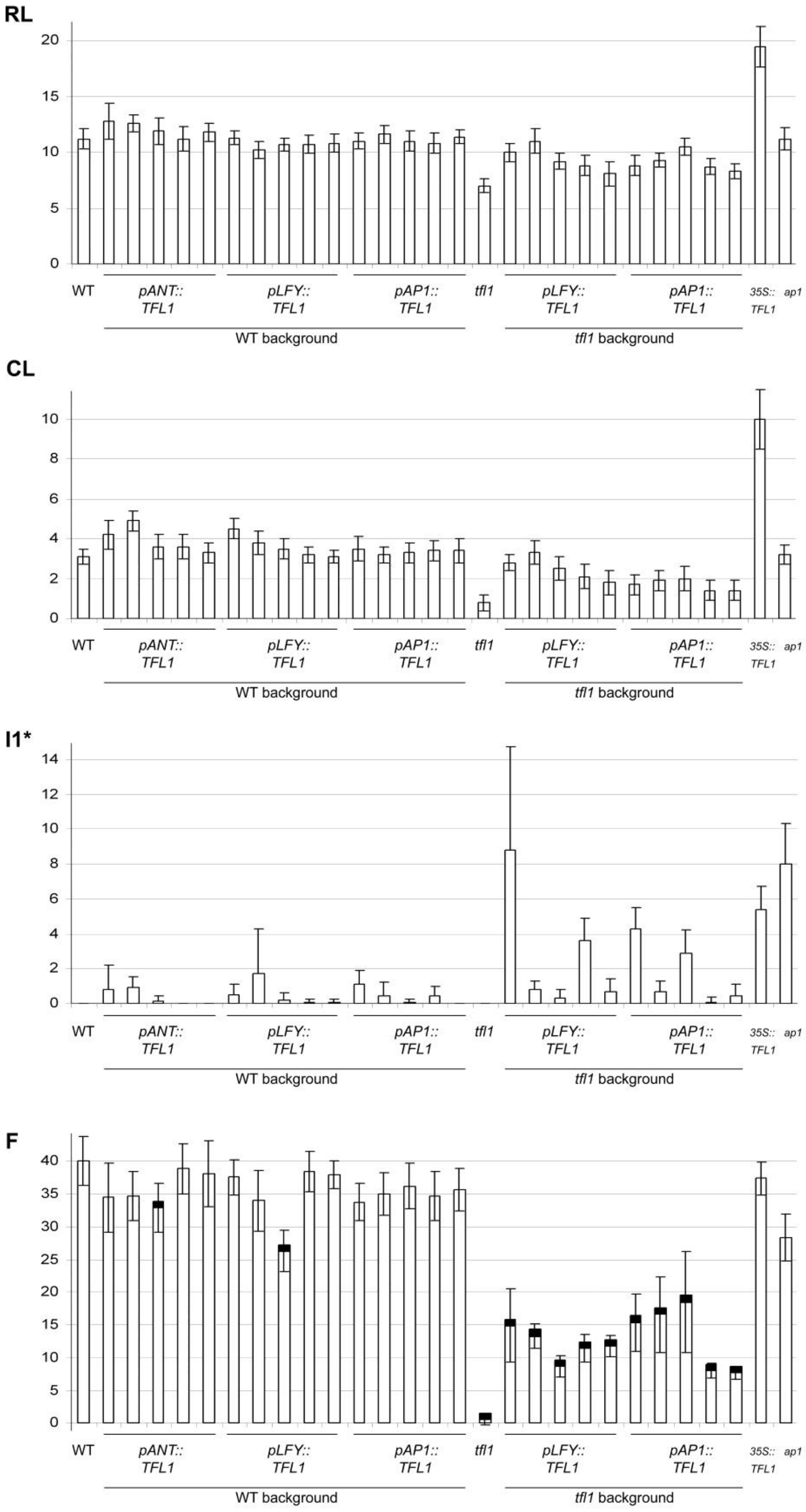

RL
CL
I1*
F
WT
pANT::
TFL1
pLFY::
TFL1
pAP1::
TFL1
tfl1
35S::
TFL1
ap1
WT background
tfl1 background
